# Comparative Study between Dermo, Pelite, and Seal-In X5 Liners: Effect on Patient's Satisfaction and Perceived Problems

**DOI:** 10.1155/2014/769810

**Published:** 2014-08-11

**Authors:** Sadeeq Ali, Noor Azuan Abu Osman, Nooranida Arifin, Hossein Gholizadeh, Nasrul Anwar Abd Razak, Wan Abu Bakar Wan Abas

**Affiliations:** Department of Biomedical Engineering, Faculty of Engineering, University of Malaya, 50603 Kuala Lumpur, Malaysia

## Abstract

*Purpose*. This study aimed to compare the effect of satisfaction and perceived problems between Pelite, Dermo with shuttle lock, and Seal-In X5 liners on the transtibial amputees. *Material and Methods*. A total of thirty transtibial amputees (17 male, 13 female) volunteered to take part in this research. Two prostheses were fabricated for each participant. Prosthetic Evaluation Questionnaire (PEQ) was filled in by the participants with the three liners. *Results*. The statistics highlight that Dermo liner showed significantly higher score (*P* = 0.05) in walking, walking on uneven surfaces, stairs walking, fitting, donning/doffing, sitting, suspension, and overall satisfaction with Dermo liner compared with Seal-In X5 and Pelite liners. Overall satisfaction was 34% higher with Dermo liner than Seal-In X5 liner and 28% higher than Pelite liner. Participants reported less problems with Dermo liner and significant differences (*P* < 0.05) were recorded between the three liners in sweating, skin irritation, frustration, and pain compared with Seal-In X5 and Pelite liners. *Conclusion*. Participants experienced high level of satisfaction and practiced fewer problems with Dermo liner. These results showed that there is good indication to believe that Dermo liner might be a good choice for transtibial users and might help the clinicians and prosthetic practitioners in selection criteria of prosthetic liners.

## 1. Introduction

Manufacturing of devices suited to individuals is a key element to recover physical capabilities. One such device is prosthesis which is aimed to substitute the loss of a limb which has lost its cosmetic and desirability for the amputee. Lower limb prosthesis can be composed of several components such as the socket, liner, shank, ankle, and foot. Among these components socket and liner are the important parts of the prosthesis due to its interface among the residuum and socket [1–3]. Poor socket fitting due to enhanced pressure between socket and residuum greatly reduces the activity level of prosthetic users [4, 5]. Amputees hold high ambulatory loading during using the prostheses in their daily activities, which is usually transferred to skeletal structure from the prosthesis via interface among residuum and prosthetic socket [6–8]. Residuum tissues are not accustomed to shear loading and skin pressure during activities. Amputee's skin is vulnerable to develop cyst, edema, dermatitis, and blisters; it is not uncommon to experience residuum skin problems in lower limb amputees [9, 10], which effect the performance and comfort of the amputee [11].

Prosthetic users required a comfortable liner and good socket to avoid skin problems and to prevent discomfort while using the prosthesis for daily activities [12, 13]. Cushioning effect of the liners lessens peak pressure and shear forces between the socket and residuum to prevent skin breakdown [14]. To make the prosthetic socket more comfortable prosthetic liners are frequently prescribed for lower limb amputees [15]. A numbers of liners are available in the market for transtibial amputees. Clinicians have been using Pelite foam liner since 1950 [16–18]. Pelite is a type of expanded cross-linked sponge foam which is shaped to fit to residuum to provide cushioning inside the socket. Many types of strategies are used to achieve a variety of suspension with Pelite liner, including suprapatellar strap or cuff or supracondylar bulge or suspension sleeve worn over the socket and extending to mid-thigh [17].

**Figure 1:**
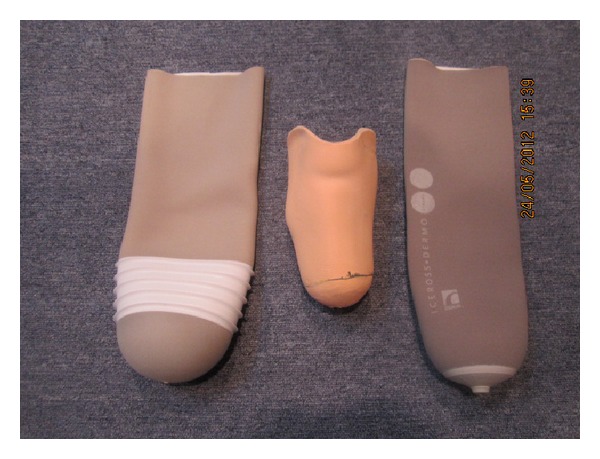


Lately, liners with superior quality have been introduced in the market. Manufacturers claim that the new liners are more comfortable with better suspension and provide relief of dermatological problems compared with previous prosthetic designs [9, 19, 20]. A wide range of liners with various properties are offered today, including the recent offering of Iceross Dermo and Seal-In X5 liners (Figure 1). Both the liners are composed of silicon material but the suspension mechanism is different. Dermo liner suspension is based on shuttle lock system, while Seal-In X5 liner has five seals around the liner for suspension conforming to the residuum shape and socket inner wall, establishing an air tight seal. Silicon liners are rolled on the patient's residuum, which enhance the contact surface with socket and provide a comfortable cushion between the prosthetic socket and residuum.

Researchers have developed many prosthetics/orthotics questionnaires to evaluate patients' satisfaction with prostheses and orthoses [20–26]. Prosthetics Evaluation Questionnaire (PEQ) is the most common type of questionnaire and majority of the researchers have mostly used PEQ to evaluate differences in performance, function, and satisfaction among different prosthetics technique or components [22, 26]. The PEQ is grouped into nine validated scales which consist of eighty-two items, and there are a number of one hundred and eleven additional individual questions pertaining to pain, satisfaction, transfer, self-efficacy, and prosthetic care. All the scales of PEQ have been validated for test-retest and internal consistency [22]. The PEQ scales are not dependent on each other, so it is reasonable to use only the scales that are pertinent to your research question. Visual analog scale format is used for PEQ questions and each line is 100 mm long and is always measured from the left (0–100) [22].

Many studies have been carried out to check the level of satisfaction and problems with transtibial liners but most of the studies are just a questionnaires survey or interview based study without fabricating prostheses for participants [27–29]. However, there is no comparative study in the literature regarding the satisfaction and perceived problems among the Pelite, Seal-In X5, and Dermo liners. Therefore this study aimed to compare the effect of satisfaction and perceived problems among Pelite, Dermo liner with pin/lock, and Seal-In X5 liner on the transtibial amputees.

## 2. Materials and Methods

### 2.1. Participants

Thirty transtibial amputees (17 male, 13 female) volunteered to take part in this research. All the participants had a unilateral amputation minimum 3 years prior to this study, who were using PTB and KBM sockets prosthesis with Pelite liner, single axis foot, and solid ankle cushioned heel (SACH) foot. The detailed particulars are shown in Table 1. University Malaya Medical Centre ethics committee approved this study, and the participants gave his/her written consent.

### 2.2. Prosthetic Intervention

A total of sixty transtibial prostheses were made up with Seal-In X5 liner with Icelock Expulsion Valve 551, Dermo liner with Icelock-200 series, socket adaptor, pylon tube, male pyramid adapter, female pyramid adapter, double adapter, and SACH foot. We fabricated two prostheses for each participant, one TSB with Dermo liner and the other TSB socket with Seal-In X5 liner. First we fabricated the prostheses with Dermo liner. Dermo liner was applied to the participant residuum properly and cellophane was applied on the liner to protect it. All the measurements and boney prominent regions were marked with transparent marker and the residuum measurements were documented. Plaster of Paris (POP) bandages were applied to residuum and massaged properly. Once the cast dried, it was removed from the participant residuum. All the marks were refreshed and negative cast was filled with POP powder. Recommended reduction was done from the positive model after removing the negative cast. Positive model was properly clean and lock was attached to the distal part of the model. Transparent plastic molding was done to get a clear socket. Clear socket was smoothed and attached with the other components. Same procedure was repeated for Seal-In X5 liner socket except that expulsion Valve was used instead of lock.

In PTB socket participants were using supracondylar suspension system and suprapatellar strap, while in TSB socket the suspension was provided through Pin/lock with Dermo liner and vacuum suspension with Seal-In X5 liner. Participant walked with the two new prostheses under the supervision of the certified prosthetist to become familiar with them. Once the participants were satisfied with the fitting, his/her next step was to use each prosthesis for at least 60 days. Participants were requested to come to the brace and limb laboratory once a week for prostheses reviews and if they required adjustment.

### 2.3. Questionnaire

In order to study the effect of the three different prosthetic liners on participant's satisfaction and to identify their problems with the use of the prosthesis, we used some elements of the PEQ. The questionnaire consists of demographic variables (sex, age, education level, marital status, height, and weight), amputation side, cause of amputation, and years since amputation. In addition, we asked some questions related to the activity levels of the participants. Four activity levels were as follows: household ambulator (K1), limited community ambulator (K2), community ambulator (K3), and high level user (K4). Medicare Functional Classification Level (MFCL) defined these levels of activities [30]. The questionnaire also included questions about participant's satisfaction and asked for details of any prosthetic-related problems that the participant experienced with each liner. In the satisfaction section of the questionnaire, participants were asked about the walking ability of the prosthesis, prosthetic fit, ability to walk up and down stairs, donning and doffing ability with their prostheses, uneven surfaces walking ability, prosthesis appearance, sitting ability with prosthesis, feeling with prosthesis, weight of the prosthesis, and overall satisfaction. Problems with the prosthesis consisted of sweating, skin irritation, wounds, pain, swelling, bad smell of residuum or prosthesis, sounds, and frustration with the prosthesis. A scale 0–100 was used to score overall satisfaction with the prosthesis, with 0 indicating that a participant was “unsatisfied” with his/her liner and 100 being indicative of “completely satisfied.” We used the same 0–100 scale of measurement for problems related variables, where 0 indicated “extremely bothered” and 100 indicated “not at all bothered.”

### 2.4. Data Collection

To avoid any mistake we explained all the questions of the questionnaire one by one to all the participants and teach them how to record your satisfaction or problems score with each prosthesis. Three separate PEQs were completed from each participant with the three different prostheses. As all the participants were using Pelite liner before the study prostheses, therefore questionnaire with Pelite liner was completed on first visit of each prosthesis before the casting for TSB sockets. After the 60-day trial period with each study prosthesis, the participants came to laboratory to complete the questionnaires for Dermo and Seal-In X5 liners prostheses to score and share his/her experience about the liners.

### 2.5. Statistical Analysis

We used nonparametric statistical analysis for data to evaluate the differences between the three liners on four main regions (anterior, posterior, medial, and lateral) and subregions (proximal and distal) of each main region. Therefore we used Kruskal-Wallis test to compare the satisfaction and perceived problems between the three liners. Analysis was performed by using version 21 of SPSS (SPSS Inc., Chicago, IL, USA) and level of significance was set at *P* < 0.05 for all analyses.

## 3. Results

The finding highlights that participants were more satisfied with Dermo liner and showed significantly higher score (*P* = 0.05) compared with Pelite and Seal-In X5 liners (see Table 2). No differences were recorded with the three liners in sitting with prosthesis, appearance of prosthesis, and weight of the prosthesis (see Table 2). Donning and doffing was significantly challenging (*P* = 0.00) with Seal-In X5 liner compared with Pelite and Dermo liners (59.00 versus 87.00 and 92.00, resp.). Overall satisfaction score was mean = 85.00, SD = 2.5 with Dermo and means = 63.00, SD = 7.91 with Seal-In X5 liner and mean = 66.00, SD = 11.25 with Pelite liner (see Table 2).

Participants experienced less sweating with Pelite liner compared with Dermo and Seal-In X5 liners (mean = 92, SD = 5.37 versus mean = 76, SD = 5.1 versus mean = 67, SD = 2.58, *P* < 0.01, resp.). More frustration, pain, and skin irritation were recorded with the Seal-In X5 and Pelite liners (see Table 2). No significant difference was observed in swelling, wound, smell, and sound with the three liners (see Table 2).

### 3.1. Comparison between Dermo and Seal-In X5 Liners

Participants showed significant (*P* < 0.05) differences between Dermo and Seal-In X5 liners in seven questions out of ten. Participants experienced 43.71% higher satisfaction during donning and doffing and 43.82% during level walking with Dermo liner compared with Seal-In X5 liners. Satisfaction was 50.34% more during feeling with the prosthesis and 29.45% higher during walking on uneven surfaces with the Dermo liner compared with Seal-In X5 liners. Overall, participants were 29.72% more satisfied with Dermo liner compared with Seal-In X5 liner. Participants noticed significantly less problems with regard to sweating (76.00 versus 67.00, *P* = 0.00), skin irritation (90 versus 83, *P* = 0.00), pain (99.00 versus 80.00, *P* = 0.00), and frustration (90.00 versus 71.00, *P* = 0.00) with Dermo liner compared with Seal-In X5 liner, respectively (see Table 3).

### 3.2. Comparison between Dermo and Pelite Liner

Participants were more satisfied with Dermo liner compared with Pelite liner and demonstrated significant (*P* < 0.05) differences during fit of the prosthesis (87.00 versus 74.00; *P* = 0.00, resp.), donning/doffing (92.00 versus 87.00; *P* = 0.05, resp.), sitting with the prosthesis (90.00 versus 83.00; *P* = 0.03, resp.), walking with prosthesis (89.00 versus 78.00; *P* = 0.00, resp.), walking on uneven surfaces (74.00 versus 66.50; *P* = 0.00, resp.), feeling with the prosthesis (92.00 versus 669.00; *P* = 0.00, resp.), and suspension with the prosthesis (88.50 versus 74.50; *P* = 0.00, resp.). Appearance and weight of the prosthesis do not show any differences (see Table 4). Overall satisfaction was 25.16% higher with Dermo liner compared with Pelite liner. Higher score was obtained with Dermo liner compared with Pelite liner during residuum skin irritation (90.00 versus 75.00; *P* = 0.00, resp.), pain (90.00 versus 70.00; *P* = 0.00, resp.), and frustration with the prosthesis (90.00 versus 71.00; *P* = 0.00, resp.). Sweating was significantly less with Pelite liner compared with Dermo liner (92.00 versus 76.00; *P* = 0.00, resp.). Sound, smell, and wound between the two liners were not statistically significant (see Table 4).

### 3.3. Comparison between Seal-In X5 and Pelite Liner

Participants were significantly satisfied with Pelite liner compared with Seal-In X5 liner during donning/doffing (59.00 versus 87.00; *P* = 0.00, resp.), walking (57.00 versus 78.00; *P* = 0.00, resp.), walking on uneven surfaces (55.00 versus 66.50; *P* = 0.00, resp.), and feeling with the prosthesis (55.00 versus 69.00; *P* = 0.00, resp.). Suspension was significantly better with Seal-In X5 liner (see Table 5). In problems part between the two liners, significantly less sweating (92.00 versus 67.00; *P* = 0.00, resp.) was recorded with Pelite liner and less pain (80.00 versus 70.00; *P* = 0.00, resp.) was observed with Seal-In X5 liner. No differences were observed in smell, wound, and swelling with the two liners (see Table 5).

## 4. Discussion

Proper fitting of socket has significant effect on patient's satisfaction, comfort, and mobility [31]. We found significant differences between the three liners both in satisfaction and perceived problems. Participant demonstrated more satisfaction and fewer problems with Dermo liner compared with Pelite and Seal-In X5 liner.

In this study, the participants favored the Dermo liner with shuttle lock over the Pelite liner and Seal-In X5 liner. These findings reflect the previous study results [27], where clear preference was given for locking liners, while in other studies Coleman et al. and Boonstra et al. showed Pelite liner to be more favorable [17, 32]. These studies oppose the findings of our research and were considerably less positive towards locking liners. The current study also mirrors the study of Ali et al. with regard to Dermo and Seal-In X5 liner [2].

Lower limb prosthesis should be functional and comfortable for the user, to give the best prospect of continued use [33]. In the study of Hatfield and Morrison [28] the participants felt more comfortable with the locking liners. Another study revealed that locking liners improved socket comfort when compared with Pelite liner [6]. In previous study the researchers also revealed that participants were more comfortable during walking and stairs negotiations with locking liners [29, 34]. The same was true with our study as the participants showed more satisfaction during walking, walking on stairs, and walking on uneven ground with the locking liner.

Skin problems are often experienced with the prostheses use in transtibial amputees and appear in the form of skin irritation, ulcers, and abrasion [35, 36]. These skin problems lead to discomfort and pain and in some cases amputees stop using the prosthesis for a period of time completely. This situation can impact satisfaction level of the amputees with prosthesis and badly affects on his/her mental health [37]. In the current research, less irritation and pain were experienced with the Dermo liner with shuttle lock compared to other liners, which mirror the studies of previous researchers [2, 29]. However, more sweating was experienced with the Dermo and Seal-In X5 liner compared with Pelite liner in our study which reflects the study of Hachisuka et al., where less sweating was reported with Pelite liner [38]. Participants feel more satisfied and experienced less pain with the Dermo liner, which leads them to walk more without any difficulties.

Fitting of socket and suspension system of prosthesis have great impacts on the participant's comfort, satisfaction, and mobility. Silicon liners are rolled over the residuum and closely attached to the skin of the residuum which creates a bond between the residuum and the liner. These qualities of the silicon liners have a positive outcome on suspension of the prosthesis [19, 20]. Two research teams revealed improved suspension with the silicon liners in their research [34, 39]. In another study researchers observed improvement in silicon liner suspension in 63% of participants compared to Pelite liner [38]. These studies mirror our results, where participants were more satisfied with Seal-In X5 and Dermo liner suspension. Many researchers recorded increase in the appearance of the prosthesis with the silicon liners which contradict the results of current study [29, 38]. In the present research participant showed the same interest in the appearance of all the three types of prostheses.

Easy donning and doffing of the prosthesis has important effect on the prosthetic users. Significant easy donning and doffing (*P* < 0.00) has revealed with the Dermo liner compared with Pelite and Seal-In X5 liner in the current study. This is same with the previous study, where the research team revealed favor donning and doffing with the locking liners [34], while in another study the researchers found both decrease and improvement [29]. The entire participants reported significant difficulties in donning and doffing with Seal-In X5 liner in this research, which might be concluded that it is due to the five seals around the liner. These results reflect the study of Ali et al., where Dermo liner showed high score for donning and doffing compared with Seal-In X5 liner.

To compare our present study results with the existing literature, it was a challenge for us as there is no study available to compare the satisfaction and perceived problems between these three liners, especially between Seal-In X5 and Pelite liner. In summary all the participants feel satisfied with the Dermo liner and revealed high performance during level walking, stairs, and uneven surfaces. The results also clarify that the participants experienced less problems and frustration with the Dermo liner.

## 5. Conclusion

The present study demonstrated that the prosthetic liners influence the level of satisfaction of transtibial users. The study results showed that Dermo liner might be the best choice for transtibial users and these results might help the clinicians and prosthetic practitioners in selection criteria of prosthetic liners. However, further study is needed with larger sample size and more detail questionnaire to comprehensively compare the effect of these three liners on amputee's satisfaction and perceived problems.

## Figures and Tables

**Table 1:** Characteristics of the participants.

Age: years, mean (SD)	46.02 ± 15.10
Sex: *n* (%)	
Male	17 (56.6%)
Female	13 (43.33%)
Weight: kg, mean (SD)	75.73 ± 14.03
Height: cm, mean (SD)	170.14 ± 6.70
Education; *n* (%)	
High school	6 (20%)
Diploma	8 (26.66%)
Degree	9 (30%)
P. Graduate	7 (23.33%)
Years since amputation: mean (SD)	7.57 ± 3.56
Reason for amputation: *n* (%)	
Diabetic	12 (40%)
Trauma	9 (30%)
PVD	5 (16.66%)
Other	4 (13.33%)
Amputation side	
Right	12 (40%)
Left	18 (60%)
Activity level: *n* (%)	
K2	7 (23.33%)
K3	6 (20%)
K4	7 (23.33%)
Prosthetics use every day: hours (SD)	9.23 ± 2.90

PVD: peripheral vascular disease.

**Table 2:** Comparison between the three liners.

Variable	Dermo	Seal-In X5	Pelite	*P* value
	Satisfaction			
Fit of prosthesis	87.00 (2.53)	78.00 (2.73)	74.00 (4.10)	0.00
Donning/doffing	92.00 (2.63)	59.00 (12.41)	87.00 (8.12)	0.00
Sitting with prosthesis	90.00 (6.70)	87.00 (4.27)	83.00 (6.32)	0.06
Walking with prosthesis	89.00 (5.17)	57.00 (12.70)	78.00 (5.37)	0.00
Walking on uneven surface	74.00 (5.70)	55.00 (8.35)	66.50 (4.74)	0.00
Walking on stairs	67.00 (5.35)	62.00 (7.17)	62.50 (5.40)	0.01
Appearance of prosthesis	87.00 (2.73)	87.00 (2.73)	85.00 (4.71)	0.51
Feeling with prosthesis	92.00 (2.73)	55.00 (11.24)	69.00 (7.10)	0.00
Weight of prosthesis	86.00 (5.47)	86.00 (5.47)	86.00 (5.16)	1.00
Suspension	88.50 (7.07)	91.00 (7.37)	74.50 (11.41)	0.00
Overall satisfaction	85.00 (2.5)	63.00 (7.91)	66.00 (11.25)	0.00

	Problems			
Sweating	76.00 (5.1)	67.00 (2.58)	92.00 (5.37)	0.00
Sound	80.00 (6.66)	82.00 (8.10)	80.00 (6.66)	0.74
Skin irritation	90.00 (0.00)	83.00 (5.41)	75.00 (7.45)	0.00
Smell	78.00 (8.11)	78.00 (7.91)	75.00 (8.81)	0.69
Wound	92.00 (8.10)	90.00 (9.42)	85.00 (9.42)	0.19
Pain	99.00 (2.10)	80.00 (3.33)	70.00 (5.77)	0.00
Frustration	90.00 (3.33)	71.00 (7.10)	71.00 (6.14)	0.00
Swelling	88.00 (2.60)	88.00 (4.21)	85.00 (4.71)	0.18

Satisfaction: 100 represented “completely satisfied” and 0 indicated “not satisfied at all.”

Problem: 100 represented “not bothered at all” and 0 indicated “extremely bothered.”

**Table 3:** Comparison between Dermo and Seal-In X5 liners.

Variable	Dermo	Seal-In X5	*P* value
	Satisfaction		
Fit of prosthesis	87.00 (2.53)	78.00 (2.73)	0.00
Donning/doffing	92.00 (2.63)	59.00 (12.41)	0.00
Sitting with prosthesis	90.00 (6.70)	87.00 (4.27)	0.23
Walking with prosthesis	89.00 (5.17)	57.00 (12.70)	0.00
Walking on uneven surface	74.00 (5.70)	55.00 (8.35)	0.00
Walking on stairs	67.00 (5.35)	62.00 (7.17)	0.03
Appearance of prosthesis	87.00 (2.73)	87.00 (2.73)	1.00
Feeling with prosthesis	92.00 (2.73)	55.00 (11.24)	0.00
Weight of prosthesis	86.00 (5.47)	86.00 (5.47)	1.00
Suspension	88.50 (7.07)	91.00 (7.37)	0.46
Overall satisfaction	85.00 (2.5)	63.00 (7.91)	0.00

	Problems		
Sweating	76.00 (5.1)	67.00 (2.58)	0.00
Sound	80.00 (6.66)	82.00 (8.10)	0.51
Skin irritation	90.00 (0.00)	83.00 (5.41)	0.00
Smell	78.00 (8.11)	78.00 (7.91)	0.87
Wound	92.00 (8.10)	90.00 (9.42)	0.63
Pain	99.00 (2.10)	80.00 (3.33)	0.00
Frustration	90.00 (3.33)	71.00 (7.10)	0.00
Swelling	88.00 (2.60)	88.00 (4.21)	0.57

Satisfaction: 100 represented “completely satisfied” and 0 indicated “not satisfied at all.”

Problem: 100 represented “not bothered at all” and 0 indicated “extremely bothered.”

**Table 4:** Comparison between Dermo and Pelite liner.

Variable	Dermo	Pelite	*P* value
	Satisfaction		
Fit of prosthesis	87.00 (2.53)	74.00 (4.10)	0.00
Donning/doffing	92.00 (2.63)	87.00 (8.12)	0.05
Sitting with prosthesis	90.00 (6.70)	83.00 (6.32)	0.03
Walking with prosthesis	89.00 (5.17)	78.00 (5.37)	0.00
Walking on uneven surface	74.00 (5.70)	66.50 (4.74)	0.00
Walking on stairs	67.00 (5.35)	62.50 (5.40)	0.00
Appearance of prosthesis	87.00 (2.73)	85.00 (4.71)	0.33
Feeling with prosthesis	92.00 (2.73)	69.00 (7.10)	0.00
Weight of prosthesis	86.00 (5.47)	86.00 (5.16)	1.00
Suspension	88.50 (7.07)	74.50 (11.41)	0.01
Overall satisfaction	85.00 (2.5)	66.00 (11.25)	0.00

	Problems		
Sweating	76.00 (5.1)	92.00 (5.37)	0.00
Sound	80.00 (6.66)	80.00 (6.66)	1.00
Skin irritation	90.00 (0.00)	75.00 (7.45)	0.00
Smell	78.00 (8.11)	75.00 (8.81)	0.64
Wound	92.00 (8.10)	85.00 (9.42)	0.08
Pain	99.00 (2.10)	70.00 (5.77)	0.00
Frustration	90.00 (3.33)	71.00 (6.14)	0.00
Swelling	88.00 (2.60)	85.00 (4.71)	0.13

Satisfaction: 100 represented “completely satisfied” and 0 indicated “not satisfied at all.”

Problem: 100 represented “not bothered at all” and 0 indicated “extremely bothered.”

**Table 5:** Comparison between Seal-In X5 and Pelite liner.

Variable	Seal-In X5	Pelite	*P* value
	Satisfaction		
Fit of prosthesis	78.00 (2.73)	74.00 (4.10)	0.02
Donning/doffing	59.00 (12.41)	87.00 (8.12)	0.00
Sitting with prosthesis	87.00 (4.27)	83.00 (6.32)	0.14
Walking with prosthesis	57.00 (12.70)	78.00 (5.37)	0.00
Walking on uneven surface	55.00 (8.35)	66.50 (4.74)	0.00
Walking on stairs	62.00 (7.17)	62.50 (5.40)	0.93
Appearance of prosthesis	87.00 (2.73)	85.00 (4.71)	0.33
Feeling with prosthesis	55.00 (11.24)	69.00 (7.10)	0.00
Weight of prosthesis	86.00 (5.47)	86.00 (5.16)	1.00
Suspension	91.00 (7.37)	74.50 (11.41)	0.00
Overall satisfaction	63.00 (7.91)	66.00 (11.25)	0.53

	Problem		
Sweating	67.00 (2.58)	92.00 (5.37)	0.00
Sound	82.00 (8.10)	80.00 (6.66)	0.51
Skin irritation	83.00 (5.41)	75.00 (7.45)	0.02
Smell	78.00 (7.91)	75.00 (8.81)	0.34
Wound	90.00 (9.42)	85.00 (9.42)	0.20
Pain	80.00 (3.33)	70.00 (5.77)	0.00
Frustration	71.00 (7.10)	71.00 (6.14)	0.87
Swelling	88.00 (4.21)	85.00 (4.71)	0.11

Satisfaction: 100 represented “completely satisfied” and 0 indicated “not satisfied at all.”

Problem: 100 represented “not bothered at all” and 0 indicated “extremely bothered.”
